# Prevalence and factors associated with Helicobacter Pylori infection among patients with dyspeptic symptoms in Tanzania: Experience from temeke regional referral hospital in Dar Es Salaam

**DOI:** 10.1371/journal.pone.0320191

**Published:** 2025-04-15

**Authors:** Phocus Novath Kibira, Malale Tungu

**Affiliations:** 1 School of Public Health and Social Sciences, Muhimbili University of Health and Allied Science, Dar es Salaam, Tanzania; 2 Jakaya Kikwete Cardiac Institute, Muhimbili National Hospital, Dar es Salaam, Tanzania; 3 Department of Development Studies, School of Public Health and Social Sciences, Muhimbili University of Health and Allied Science, Dar es Salaam, Tanzania; Addis Ababa Science and Technology University, ETHIOPIA

## Abstract

**Introduction:**

*Helicobacter pylori* is a type of bacteria that infects the stomach lining and can cause various gastrointestinal disorders, such as dyspepsia, gastritis and peptic ulcers. Dyspepsia is a common symptom among patients seeking medical care, and *Helicobacter pylori* infection is one of the most common causes of dyspepsia.

**Objective:**

This study aimed to determine the prevalence and factors associated with *Helicobacter pylori* infection among patients attending Temeke Regional Referral Hospital (RRH) in Dar es Salaam, Tanzania.

**Methods:**

A hospital-based cross-sectional study involving dyspeptic patients was conducted between May and June 2023. A Standardized data collection tool was used to collect socio-demographic characteristics and other information such as level of income and source of water. *Helicobacter pylori* antigen was detected using a stool *Helicobacter pylori* antigen rapid test according to the manufacturer’s instructions. Data analysis was done using STATA 15 computer software.

**Results:**

The study revealed that the prevalence of *Helicobacter pylori* infection among dyspeptic patients was 43.77%. Also, male sex, occasional hand washing habits and participants who were not sure whether they used treated water for drinking were the factors that were strongly associated with positive *Helicobacter pylori* infection.

**Conclusion:**

Preventive measures and eradication of *Helicobacter pylori* infection should be considered worthy by public health authorities. More studies have to be emphasized to check the relationship between sex and *Helicobacter pylori* infection.

## Introduction

*Helicobacter pylori* is a type of bacteria that infects the stomach lining and can cause various gastrointestinal disorders, such as dyspepsia (indigestion), gastritis (inflammation of the stomach lining), and peptic ulcers [1–3]. Dyspepsia is a common symptom among patients seeking medical care and *Helicobacter pylori* infection is one of the most common causes of dyspepsia [4,5]. It causes a chronic infection with can lead to lifelong inflammation of gastric mucosa [6]. In some individuals, gastric atrophy develops into gastric cancer, while in others it may lead to duodenal ulcer. Gastritis, peptic ulcer disease, and gastric cancer are primarily caused by *Helicobacter pylori* infection [7].

In most cases, a childhood *Helicobacter pylori* infection can last a lifetime without receiving antibiotic treatment. Most infected people don’t exhibit any symptoms for a long time [8]. Consequently, chronic *Helicobacter pylori* colonization can harm the gastric mucosa and result in several diseases of the upper gastrointestinal tract [9,10]. According to studies, *Helicobacter pylori* infection causes 89% of all cases of stomach cancer, and its eradication minimizes the risk of the disease [11,12]. Statistics from 2018, the second cause of cancer-related fatalities worldwide and the sixth most common cancer overall is gastric cancer [13].

The necessity to control and eradicate *Helicobacter pylori* infection is still crucial to continue lowering the burden of gastric cancer despite the fact that gastric cancer incidences and deaths have considerably dropped over the past 20 years in the majority of countries globally [14,15]. In high prevalence communities, it has been proven that *Helicobacter pylori* screening and eradication are more cost-effective ways to lessen the burden of stomach cancer and peptic ulcers [2]. A current understanding of the prevalence of *Helicobacter pylori* and its contributing factors is necessary to develop effective *Helicobacter pylori* eradication strategies. Moreover, *Helicobacter pylori* infection has been linked to a number of extra-digestive illnesses [16]. The most likely *Helicobacter pylori* transmission routes are feco-oral, oro-oral, and intra-familial pylori are all common [17]. Food and personal cleanliness are intimately linked to risk factors for *Helicobacter pylori* infection. Additional known risk factors for *Helicobacter pylori* infection include age, socioeconomic position, the number of siblings, crowding in the home, ethnicity, migration from areas with high incidence, family member infection status, and access to sanitary facilities [18–20]. *Helicobacter pylori* can be diagnosed using invasive and non-invasive techniques [21–24]. Invasive tests have a key drawback in that they require an endoscopic examination to get a diagnostic sample, making it challenging to employ them in epidemiological investigations. The *Helicobacter pylori* stool antigen test (HpSAT) has significantly advanced in recent years and has attracted a lot of attention for *Helicobacter pylori* detection [25,26].

Between 85% and 95% of people in low- and middle-income countries (LMICs) have *Helicobacter pylori*, compared to between 30% and 50% of people in developed nations [27–29]. With advancements in sanitation and eradication techniques, the epidemiology of *Helicobacter pylori* infection has changed. Nonetheless, *Helicobacter pylori* is still widely prevalent. Prevalence is still high in poor countries and this is correlated with socioeconomic position and standards of hygiene. Switzerland had the lowest reported frequency (18.9%), while Africa had the greatest recorded prevalence (70.1%) [30].

It was useful to comprehend the prevalence of *Helicobacter pylori* in Dar es Salaam and its associated determinants to prioritize and customize public health measures to better manage the burden of *Helicobacter pylori* infections and its associated disorders. There has been a report of rising triple therapy antimicrobial resistance, hence *Helicobacter pylori* remains a big challenge to the health sector and a major public health problem.

Hence, a number of methods, such as sequential, concurrent, and hybrid therapies are suggested to raise the success rate of first-line therapy for *Helicobacter pylori* infection. Thirty years after its discovery, *Helicobacter pylori* infection still poses a significant challenge to all gastroenterologists because the optimum first-line eradication regimen with the highest eradication rate and minimal side effects is still unknown, and the precise mode of transmission is still unknown [5].

The identification of factors associated with *Helicobacter pylori* infection in this population is crucial to guide effective preventive and management strategies. This study aimed to determine the prevalence and factors associated with *Helicobacter pylori* infection among patients with dyspeptic symptoms at Temeke RRH in Dar es Salaam, Tanzania. The results of the study would fill the knowledge gap and provide important insights into the burden of *Helicobacter pylori* infection and inform strategies for its prevention and management.

## Materials and methods

### Study design

A hospital-based cross-sectional study was conducted at Temeke RRH using a quantitative approach to analyze the existence of the infection with *Helicobacter pylori.*

### Study area

The research was conducted from May to June 2023 at Temeke RRH. Tanzania had 336 hospitals in total as of 2022. With 53 hospitals, Dar es Salaam has the most of any region [31]. Temeke district has a 240 square kilometer land area and a 5-kilometer-long coastal zone, and is the largest municipality in Dar es Salaam. In addition, it is 390 12 degrees’ - 390 33’ East and 60 48–70 33’ South. Temeke is situated south of the city of Dar es Salaam, where the north-west is bordered with Ilala district, the north-east is bordered with Kigamboni district, while the south-east borders Mkuranga district of Coast Region, and in the east borders the Indian Ocean. In 2019, it is anticipated that the population will increase to 2,367,578 according to the population census projection methodology. The municipal population was 1,205,949 as per the Population and Housing Census in 2012, with 618,092 females and 587,857 males making up the gender distribution. It is anticipated that in 2019, there will be 1,154,109 males and 1,213,468 females [32,33].This study involved Temeke RRH, as it is the largest public hospital in the district and has the highest referral level. It is meant to serve the entire population of Temeke in the pyramidal referral system [31].

### Study population

All patients with dyspeptic symptoms, above 18 years of age, attended Temeke RRH.

### Exclusion and inclusion criteria

#### Inclusion criteria.

- Patients who presented with dyspeptic symptoms, such as epigastric pain, heartburn, nausea, vomiting, bloating, or early satiety.
- All patients who had dyspeptic symptoms above 18 years of age attending at Temeke RRH. The prevalence of *Helicobacter pylori* infection can vary by age. Excluding children allowed us to focus on a specific adult population with a higher likelihood of infection.
- Patients who had the will to provide informed consent and participate in the study.

#### Exclusion criteria.

- Patients who were unable to provide informed consent or complete study procedures, such as those with cognitive impairment or language barriers.
- Critically ill patients who had other comorbidities.

### Sampling technique

A purposive sampling technique was used to determine the area of study among 5 Municipal Councils in Dar es Salaam, in the context of this study. Temeke Municipal Council was purposively selected because most of the houses are overcrowded, with low hygiene, poor sanitation and poor drainage systems, hence which were postulated to increase the risk of *Helicobacter pylori* infection. Temeke RRH was selected due to serving a huge number of population compared to other public health facilities in the municipal[31,33]. Purposive sampling of patients with dyspeptic symptoms was employed by the doctors attending patients at the Out-Patient Department (OPD) clinic through serial enrollment.

### Data collection methods

Data about the independent variables (level of income, hand washing hygiene, water treatment, and source of drinking water) and the social demographic information were obtained from the patients by using a questionnaire after informed consent. The data collection tool was an open source mobile data collection platform (ODK). The *Helicobacter pylori* stool antigen rapid test results for the patients who were recruited to the study were obtained from the Hospital information management system (EHMS) from May to June 2023. *Helicobacter pylori* stool antigen rapid test has sensitivity and specificity of 100% and 92% respectively, therefore it is a good alternative for diagnostic tests such as the urea breath test [26].

### Data analysis

Statistical analysis was performed using Stata version 15. The data were summarized and tabulated to generate social demographic characteristics (age, gender and location), level of income, hand washing hygiene and water treatment. Descriptive analysis: the data were summarized using descriptive statistics such as mean, median, mode, standard deviation, and frequency distribution. Prevalence analysis was done by dividing the number of patients with a positive *Helicobacter pylori* test result by the total number of dyspeptic patients tested, in the descriptive analysis.

Logistic regression analysis: This involved examining the relationship between the independent variables (such as age, sex, occupation, etc.) and the dependent variable (*Helicobacter pylori* infection status) using Stata version 15. The associations of the demographic characteristics and other factors, with the *Helicobacter pylori* infection were statistically significant when the P value was less than 0.2 (95% confidence interval (CI)) in the bivariate logistic regression and less than 0.05 (95% CI) in the multivariate logistic regression analysis.

### Ethical consideration

Ethical clearance was obtained from the Muhimbili University of Health and Allied Sciences (MUHAS research review board) in May 2023 (MUHAS-REC-05-2023-1670). Following a full description of the purpose and benefit of the study, permission to perform it was obtained from the Regional Administrative Secretary, Regional Medical Officer, Municipal Director, and District Medical Officer. Participants were duly informed of the purpose of the study and their rights. Written informed consent for this study that includes data collection and consent to participate was requested and obtained from the participants and they were assured of their anonymity in publications. All methods were carried out in accordance with relevant guidelines and regulations of the approval bodies and in accordance with the Declaration of Helsinki.

## Results

### Prevalence of *Helicobacter pylori* infection

**Fig 1:**
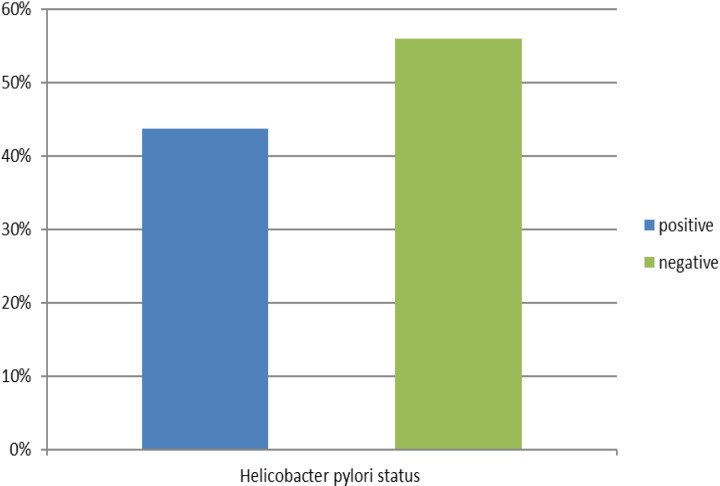
Shows the prevalence of Helicobacter pylori infection among patients with dyspeptic symptoms at Temeke RRH.

A total of 393 patients with dyspeptic symptoms participated in the study. Among them, 221 patients (56.2%) tested negative for *Helicobacter pylori* infection, while 172 patients (43.8%) tested positive. The *Helicobacter pylori* positive cases were 170 among a total of 393 participants, revealing a prevalence of 43.8% as indicated in Figure 1.

### Socio-demographic characteristics

As shown in Table 1 above, 393 patients with dyspeptic symptoms who were recruited at Temeke RRH OPD clinic. The patients’ ages ranged from 18 to 60 years old. The age range of the majority of individuals (30.79%) was found to be between 40 and 49 years old. Interestingly, this age group also constituted the majority of those with *Helicobacter pylori* positive status (29.65%). The bulk of study participants (51.7%) were female, however, the male population had a higher prevalence of *Helicobacter pylori* infection, accounting for up to 59.3% of cases. 280(71.2%) and 113(28.8%) of the 393 participants lived in the Temeke district and outside of it, respectively. Of the 172 positive instances, Temeke Municipal Council accounted for 126 (73.26%), or the majority of the positive cases among all districts. The group from Kinondoni had the fewest participants overall (3.05%).

**Table 1:** Socio-demographic characteristics of participants stratified by *Helicobacter pylori* status (N = 393).

	Positive	Negative	Total (%)
Sex			
Male	102(59.3%)	88(39.82%)	190 (48.35%)
Female	70(40.7%)	133(60.18%)	203 (51.65%)
Total	172(100%)	221(100%)	393(100%)
Age			
18-29 years	33 (19.19%)	47 (21.27%)	80 (20.36%)
30-39 years	45 (26.16%)	51 (23.08%)	96 (24.43%)
40-49 years	51 (29.65%)	70 (31.67%)	121 (30.79%)
50-59 years	30 (17.44%)	30 (13.57%)	60 (15.27%)
60 years or older	13 (7.56%)	23 (10.4%)	36 (9.16%)
Total	172(100%)	221(100%)	393(100%)
Residence			
Temeke	126 (73.26%)	154 (69.68%)	280 (71.25%)
Kinondoni	5 (2.91%)	7 (3.17%)	12 (3.05%)
Ubungo	8 (4.65%)	20 (9.05%)	28 (7.12%)
Kigamboni	10 (5.81%)	12 (5.43%)	22 (5.6%)
Ilala	23 (13.37%)	28 (12.67%)	51 (12.98%)
Total	172(100%)	221(100%)	393(100%)

### Characteristics of the Respondents

As shown in Table 2, the study included 393 patients with dyspeptic symptoms who were recruited at the OPD clinic at Temeke RRH, with ages ranging from 18 years to 60 years or older. It was observed that the majority of the participants had ages ranging from 40–49 years (30.79%). it’s also this age group that turned to form the majority, among the participants with *Helicobacter pylori* positive status (29.65%). *Helicobacter pylori* positive and negative male participants were 102 and 88 respectively. Female 203 (51.7%) formed the majority of the study population, although the male population was more infected with *Helicobacter pylori* infection, setting up 59.3% of the participants who were *Helicobacter pylori* positive. Of 393 participants; 280 (71.2%) and 113(28.8%) were residing in Temeke district and outside Temeke district respectively. The least number of total participants were from Kinondoni (3.05%), this may be due to the distance factor between Temeke RHH and Kinondoni municipal.

**Table 2:** Socio-demographic characteristics of participants and factors associated with *Helicobacter pylori* infection, stratified by *Helicobacter pylori* status (N = 393).

	Male	Female	Total (%)
Positive	102 (53.68%)	70 (34.48%)	172(43.77%)
Negative	88 (46.32%)	133 (65.52%)	221(56.23%)
Total	190 (100%)	203 (100%)	393 (100%)
Age			
18-29 years	29 (15.26%)	51 (25.12%)	80 (20.36%)
30-39 years	51 (26.84%)	45 (22.17%)	96 (24.43%)
40-49 years	60 (31.58%)	61 (30.05%)	121 (30.79%)
50-59 years	30 (15.79%)	30 (14.78%)	60 (15.27%)
60 years or older	20 (10.53%)	16(7.88%)	36 (9.16%)
Total	190 (100%)	203 (100%)	393 (100%)
Residence			
Temeke	144 (75.79%)	136 (67%)	280 (71.25%)
Kinondoni	2 (1.05%)	10 (4.93%)	12 (3.05%)
Ubungo	10 (5.26%)	18 (8.87%)	28 (7.12%)
Kigamboni	12 (6.32%)	10 (4.93%)	22 (5.6%)
Ilala	22 (11.58%)	29 (14.29%)	51(12.98%)
Total	190 (100%)	203 (100%)	393 (100%)
Water treatment			
Treated	101 (53.16%)	116 (57.14%)	217 (55.22%)
Not treated	74 (38.95%)	75 (36.95%)	149 (37.91%)
Not sure	15 (7.89%)	12 (5.91%)	27 (6.87%)
Total	190 (100%)	203 (100%)	393 (100%)
Hand washing			
Rarely	46 (24.21%)	35 (17.24%)	81 (20.61%)
Occasionally	117 (61.58%)	143 (70.44%)	260 (66.16%)
Frequently	27 (14.21%)	25 (12.32%)	52 (13.23%)
Income level			
Unemployed	76 (40%)	109 (53.69%)	185 (47.07%)
Employed	114(60%)	94 (46.31%)	208 (52.93%)
Total	190 (100%)	203 (100%)	393 (100%)

55.22% of total participants, reported using treated water for drinking, 37.91% reported the used untreated water for drinking, while 6.87% were not sure if they used treated or untreated water for drinking. Among the *Helicobacter pylori* positive cases, the majority reported having used treated water for drinking 48.84%, 40.70% of them reported using untreated water, while some of them were not sure if water for drinking was treated or not.

47.07% of the participants reported being unemployed. Among positive total cases, the unemployed participants formed the majority of the *Helicobacter pylori* positive cases (50.58%). Also, among the negative *Helicobacter pylori* cases, the majority of the population was formed by employed individuals (55.66%). As for hand washing hygiene,66.16% of the participants reported washing their hands with soap occasionally, while 20.61% and 13.23% washed reported behaviour of washing their hands with soap rarely and frequently respectively. Also, among positive *Helicobacter pylori* cases, 58.14% reported washing their hands with soap occasionally, 27.91% washed their hands rarely with soap, while 13.95% washed their hands frequently with soap.

### Regression analysis

From Table 3, we observed that, sex was statistically significantly associated with positive *Helicobacter pylori* status; male participants were 2.2 times more likely to acquire *Helicobacter pylori* infection than female participants (OR=2.20; 95% CI [1.47, 3.31]). The findings indicate that those who washed their hands occasionally and frequently are more likely to reduce the possibility to be infected than those who washed their hands rarely (OR=0.43; 95% CI [0.26, 0.71]).

**Table 3:** Univariate and multivariate logistic regression analysis of factors associated with *Helicobacter pylori* infection among 393 adult patients with dyspeptic symptoms.

Independent variables	Positive test (N, %)	Univariate analysis		Multivariate analysis	
		OR (95% CI)	P value	OR (95% CI)	P value
Age(n)					
18-29 years (80)	33(41.25)	1		1	
30-39 years (96)	45(46.88)	1.26(0.69- 2.29)	0.455	1.18(0.59-2.35)	0.641
40-49 years (121)	51 (42.15)	1.04(0.59 -1.84)	0.899	0.95(0.49-1.87)	0.890
50-59 years (60)	30(50.00)	1.42 (0.73-2.79)	0.304	1.32(0.60-2.87)	0.489
60 years or older (36)	13(36.11)	0.81(0.36-1.81)	0.601	0.44(0.16-1.19)	0.106
Sex					
Female (203)	70(34.48)	1		1	
Male (190)	102(53.68)	2.20 (1.47-3.31)	0.000	2.16(1.38-3.38)	0.001
Residence					
Temeke (280)	126(45.00)	1		1	
Kinondoni (12)	5(41.67)	0.87 (0.27-2.82)	0.820	1.17(0.33-4.10)	0.804
Ubungo (28)	8(28.57)	0.49 (0.21-1.15)	0.100	0.57(0.22-1.48)	0.248
Kigamboni (22)	10(45.45)	1.02 (0.43-2.44)	0.967	0.84(0.30-2.35)	0.744
Ilala (51)	23(45.10)	1.00 (0.55-1.83)	0.990	0.87(0.45-1.71)	0.689
Water treatment					
Treated (217)	84(38.71)	1		1	
Not treated (149)	70(46.98)	1.40(0.92-2.14)	0.116	1.11(0.69-1.85)	0.682
Not sure (27)	18(66.67)	3.17(1.36-7.38)	0.008	3.97(1.55-10.21)	0.004
Hand washing					
Rarely (81)	48(59.26)	1		1	
Occasionally (260)	100(38.46)	0.43(0.26-0.71)	0.001	0.41(0.23-0.73)	0.002
Frequently (52)	24(46.15)	0.59(0.29-1.19)	0.140	0.65(0.30-1.41)	0.275
Income level					
Unemployed (185)	87 (47.03)	1		1	
Employed (208)	85 (40.87)	0.78 (0.52-1.16)	0.219	0.69(0.40-1.18)	0.174

For those who used untreated water, had a chance of being infected with *Helicobacter pylori* by 40% compared to those who used treated water (OR=1.40; 95% CI [0.92, 2.14]), Also those who were not sure of the water treatment were three times far more probable to contract *Helicobacter pylori* in contrast to those who used treated water (OR=3.17; 95% CI [1.36, 7.38]).

Age, employment status, source of drinking water and Residence of the participants were not statistically significantly associated with positive *Helicobacter pylori* status among participants.

## Discussion

This study found a high prevalence of *Helicobacter pylori* infection status which was close to half among dyspeptic patients who participated in the study, among dyspeptic patients was 43.8%. However, the prevalence rate shown in our study is lower than the average global prevalence rate (50%) [27,34] and most African reviews [34,35]. The prevalence rate found in our study was slightly higher compared to the findings of the previous study done in Mwanza by Jaka, Hyasinta [5], The disparity in prevalence rates between these two parts of the country may largely be linked to the overcrowding in the Temeke Municipal Council and low hygiene standards [5,34]. Low socioeconomic level has been linked to increased *Helicobacter pylori* prevalence and *Helicobacter pylori* transmission. Previous studies indicate that domicile overcrowding, high housing density, as well as sharing of beds, particularly in developing countries, have all been linked to domestic infection, and it has been hypothesized that these factors contribute to the spread of the infection [5,7].

In our study, males had a higher prevalence of *Helicobacter pylori* than females, with a female preponderance. Many populations have a variety of gender effects on the prevalence of *Helicobacter pylori* infection. This disparity could be due to variations in lifestyle, dietary habits, or other factors that contribute to *Helicobacter pylori* transmission and infection. The findings of this study concur with a study which was conducted in Mwanza, which revealed that there was an insignificant correlation between sex and *Helicobacter pylori* infection [5]. In the United Arab Emirates (UAE) study, female participants were shown to be more likely to have *Helicobacter pylori* infection, presumably as a result of the fact that the majority worked as nannies and came from households with poor socioeconomic standing [7]. A study done in Iran revealed that there was no association was detected between *Helicobacter pylori* positivity and gender [36].

Another finding was the source of drinking water whereby the study found that participants who used tap water had a higher likelihood of contracting *Helicobacter pylori* in contrast to those who used other water sources. This might be due to the fact that there is a possibility of *Helicobacter pylori* transmission through contaminated tap water. Studies conducted in Tanzania [5] and UAE [7], revealed that untreated drinking water is one of the significant factors in contaminating *Helicobacter pylori* infection [7]. A significant proportion of participants reported using untreated water for drinking, which may pose a potential risk for *Helicobacter pylori* infection [5,37,38]. However, a majority of *Helicobacter pylori* positive cases reported using treated water for drinking, indicating that the transmission route may differ from the water supply system. Numerous researches have already proved that water is a significant way for *Helicobacter pylori* to spread [7,5,38]. So, improper water handling and bad sanitation will be conducive to the spread of this ailment.

The majority of participants reported washing their hands with soap occasionally. Those who had no habit of washing their hands were more likely to acquire *Helicobacter pylori* infection compared to those who washed their hands occasionally and frequently. This may be due to the reason that by implementing household hygiene practices, such as hand washing, the risk of acquiring and transmitting *Helicobacter pylori* can be significantly reduced and prevented. A study done in South Western Uganda found that the risk of contracting *Helicobacter pylori* infection escalated when there was no hand washing station available [37]. These findings suggest that there may be room for improvement in hand hygiene practices, as inadequate hand hygiene can contribute to the transmission of *Helicobacter pylori*. These findings concur with the previous studies which indicated that improving the overall population’s sanitary conditions such as hand washing hygiene can prevent *Helicobacter pylori* acquisition [5,7,35,36,38].

### Strengths and limitations of the study

This study merely gave a general summary of *Helicobacter pylori* prevalence in Tanzania. It is regarded as a pilot study and as a result of its limitations, such as the small sample size. The findings cannot be extrapolated to the entire Tanzanian population. More initiatives should be made to carry out additional multicenter, large-scale epidemiological investigations over all of Tanzania. Also, other studies can conduct a qualitative study to probe the insights of the prevalence of *Helicobacter pylori* among patients. The strength of the study could lie in its potential to provide valuable insights into the prevalence of *Helicobacter pylori* among patients with dyspeptic symptoms, shedding light on its epidemiology and associated factors. This can aid in better understanding the burden of *Helicobacter pylori* infection and inform clinical management strategies for dyspepsia.

### Conclusion and reccomendations

This study revealed a significant prevalence of *Helicobacter pylori* infection among patients with dyspeptic symptoms, indicating a substantial burden in the study area. Recommendations include implementing health education programs emphasizing hand hygiene, improving access to safe water sources, and establishing routine screening programs at primary healthcare centers. Additionally, further research is needed to understand the impact of gender on infection rates and to explore additional risk factors contributing to *Helicobacter pylori* transmission. Continuous surveillance is also advised to monitor prevalence, trends, and antibiotic resistance patterns in the region.

## Supporting information

“Supporting Information files xlsx” includes data of the study about the Prevalence and Factors Associated with Helicobacter Pylori Infection among Patients with Dyspeptic Symptoms in Tanzania: Experience from Temeke Regional Referral Hospital in Dar es Salaam. (XLSX)

## List of Abbreviations

CI: Confidence Interval
EHMS: Electronic Health Management System
HpSAT: Helicobacter pylori stool antigen test
ODK: Open Data Kit
OPD: Out- Patient Department
OR: Odds Ratio
RRH: Regional Referral Hospital
UAE: United Arab Emirates.
